# The hemoglobin, albumin, lymphocyte, and platelet (HALP) is a potent indicator for the prognosis in hemodialysis patients

**DOI:** 10.1097/MD.0000000000033650

**Published:** 2023-05-12

**Authors:** Fengping Zhang, Luohua Li, Taotao Shi, Yu Liu, Jun Xie, Le Yu

**Affiliations:** a Department of Nephrology, Jiujiang No.1 People’s Hospital, Jiujiang, China; b Department of Nephrology, West China Hospital, Sichuan University, Chengdu, China; c Department of Nephrology, Pingxiang People’s Hospital, Pingxiang, China; d Department of Nephrology, De ‘an People’s Hospital, Jiujiang, China; e Department of Rheumatology and Immunology, Jiujiang No.1 People’s Hospital, Jiujiang, China.

**Keywords:** HALP, hemodialysis, prognosis, risk factors

## Abstract

The hemoglobin, albumin, lymphocyte, and platelet (HALP) values were marked as a original index of general nutritional and inflammatory conditions. The purpose of this investigation was to evaluate the potential relationship between HALP and prognosis in hemodialysis (HD) patients. Patients with maintenance HD from multiple dialysis centers in China were retrospectively analyzed. The primary poor outcome were cardiovascular disease (CVD) and all-cause death. The computational equation of HALP values as the follows: hemoglobin (g/L)** × **albumin (g/L) **×** lymphocytes (/L)**/** platelets (/L). All participants were divided into Tertile 1, Tertile 2, and Tertile 3 according to the tertiles of baseline HALP values. The Kaplan–Meier curve and the Cox regression was done to figure out the relationship about HALP and adverse outcomes. The restricted cubic splines further identified the possible associations. The time-dependent receiver operating characteristic curve and C-index were implemented for evaluate the predictive values of the HALP composite model. There were 4796 patients incorporate into ultimate study. Compared with patients in Tertile 1, patients in Tertile 3 had an lower risk of all-cause mortality [hazard ratios = 0.66, 95% confidence intervals: 0.49–0.86, *P* = .007] and CVD mortality [sub-distribution hazard ratio = 0.51, 95% confidence intervals: 0.34–0.80, *P* = .005]. The composite model with the supplement of HALP outperformed the traditional factor model in the time-dependent receiver operating characteristic curve. High HALP values at baseline are related to a diminished risk of CVD death and all-cause death in HD patients. HALP is a novel and potent index for the prognosis of HD patients.

## 1. Introduction

And indeed, hemodialysis (HD) remains the greatest frequent constituent part of kidney replacement therapy in patients with end-stage kidney disease, composing around 89% of overall dialysis and 69% of kidney replacement therapy. Despite continuous innovation in dialysis technology and quality of care, the burden of symptoms, economics and mortality remains grave, notably in low and middle-income countries. Essentially 67% of HD dialysis is influenced by cardiovascular disease (CVD), accounting for nearly 50% of mortality, and is the key content of standardized outcomes in nephrology-HD.^[1,2]^ Previous studies have proved that immune disorders, chronic inflammation and nutritional deficiencies are important risk factors leading to adverse outcomes of patients, which are intimately related to the occurrence of CVD matters and all-cause death.^[3,4]^ However, the commonly used clinical prognostic indicators have not been fully verified (such as blood phosphorus level, blood calcium level, weekly adequacy of dialysis, hemoglobin, micro-inflammatory biomarkers, and oxidative stress).^[5,6]^ Studies have reported that these indicators are related to poor outcomes of HD patients, but there are large differences in these studies. The single index has low predictive ability and 1-sided reflection of body characteristics. Finding a unified definition, convenient collection, and comprehensive reporting of evaluation systems is essential to ameliorate the survival in HD patients.

The hemoglobin, albumin, lymphocyte, and platelet (HALP) composite values were acknowledged as a original index of general nutritional and inflammation condition.^[7,8]^ and has recently been identified as bladder cancer, breast cancer, acute heart failure, acute ischemic stroke, and other important prognostic factors in patients.^[9–13]^ Nevertheless, it is indefinite if HALP values are correlated with prognosis in HD patients, and our work aimed to determine the association of HALP with all-cause mortality and CVD mortality.

## 2. Subjects and methods

### 2.1. Subjects

Research participants were collected from the clinical database of dialysis and follow-up in 4 large hemodialysis centers in China, which is located on the website of Chinese national renal data system (www.cnrds.net). In this study, we retrospectively analyzed the patients with maintenance HD over 18 years of age and dialysis duration more than 3 months from January 2008 to October 2022. The baseline period for inclusion was 1 week before the start of dialysis. The exclusion criteria were as follows: Have been recent invasive operations except surgery for dialysis access; With acute infection, autoimmune disease, malignant tumor or hemopathy; The use of anti-inflammatory, antiplatelet or immunosuppressive drugs; Missing essential baseline data such as blood cell count and serum albumin; and Without outcome data. The Ethics Review Committee approved the study programme (JJSDYYMYY-YXLL-2022-108), and the patient’s informed consent form was exempted because it was a retrospective data analysis and there was no individual identification information.

### 2.2. Methods

#### 2.2.1. General data

Demographic and clinical factors included age, gender, body mass index, smoking status, comorbidities (including diabetes, hypertension and past history of CVD), and common drug use during the baseline period of the analysis. Laboratory values included blood cell count (hemoglobin, neutrophil count, lymphocyte count, monocyte count, platelet count), biochemical indicators (alanine transaminase, aspartate transaminase, albumin, blood lipids, uric acid, serum creatinine, estimated glomerular filtration rate (eGFR), blood calcium, blood phosphorus), intact parathormone (iPTH) and C-reactive protein (CRP).

#### 2.2.2. HALP

The baseline blood count data was used to evaluate the markers needed for the study. The computational equation of HALP values as the follows: hemoglobin (g/L)** × **albumin (g/L) **×** lymphocytes (/L)**/**platelets (/L).

#### 2.2.3. Clinical end points

The end points measure of the study were CVD death and all-cause death. CVD death included cardiac failure, coronary heart disease, pulmonary edema, stroke, arrhythmia, and other fatal CVD status. The determination of cause of death was obtained after a clinical case discussion.

#### 2.2.4. Statistical processing

The conforming to normal distributions were displayed as mean ± standard deviation, while skewed distributions were expressed as median (interquartile range: P25, P75). Classification data were shown as frequency (n) and percentage (%). Derived from the tertiles of baseline HALP values, the object of observation was chopped up into 3 groups: Tertile 1, Tertile 2, and Tertile 3. The cumulative risk of death in the baseline HALP values were determined by the Kaplan–Meier curve and log-rank analysis. The Cox regression was done to figure out the risk of adverse outcomes in the ternary of HALP. Frequently reported confounding factors that involved in the causes of CVD and all-cause death for HD were considered to be potential covariates. Time-dependent receiver operating characteristic curve and C-index were conducted in estimate the prognostic value about HALP and compared with traditional factors. Besides, we implemented restricted cubic splines to study the potential nonlinear relationship between HALP continuous variables with the hazard of all-cause mortality. R software (www.r-project.org) was applied to analyze in this research. Significance was defined at *P* value < .05.

## 3. Results

### 3.1. Baseline characteristics

**Figure 1.**
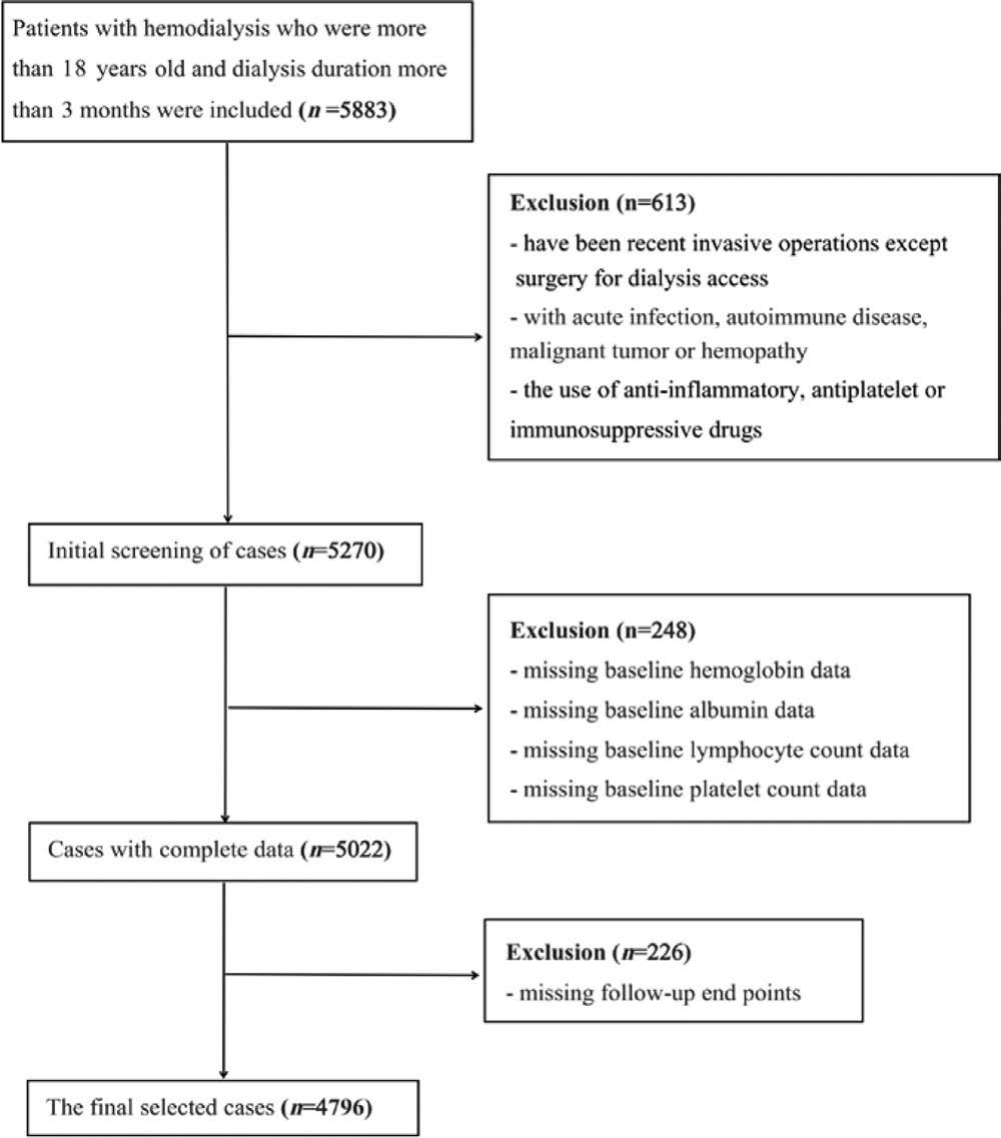
Flow chart for recruitment of cases.

We selected 5883 participants and finally analyzed the available population data of 4796. The flow map was shown in Figure 1. The average age of all patients was 55.8 ± 12.3 years old, including 2226 (46.4%) males and 2570 (53.6%) females. The baseline HALP values were 26.4 (17.1, 35.2). According to the baseline data range, the subjects were divided into 3 groups: Tertile 1 (<20.3, n = 1599), Tertile 2 (≥20.3, ≤31.6, n = 1599) and Tertile 3 (<31.6, n = 1598). The results indicated that: in contrasted to patients with increased HALP, patients with decrease HALP had older age, more diabetes and a higher proportion of previous history of CVD; lower lymphocyte count, monocyte count, hemoglobin, albumin, and calcium levels; higher neutrophil count, platelet count, blood phosphorus and CRP levels; and other demographic characteristics and clinic parameters of the participants were shown in Table 1.

**Table 1:** Comparison of general baseline data in different groups of hemoglobin, albumin, lymphocyte, and platelet (HALP).

	Total (n = 4796)	Tertile 1 (n = 1599)	Tertile 2 (n = 1599)	Tertile 3 (n = 1598)	*P* value
Age (yr)	55.3 ± 12.3	57.6 ± 11.9	54.4 ± 12.2	53.5 ± 12.1	<.001
Male, *n* (%)	2226 (46.4)	735 (46.0)	742 (46.4)	749 (46.9)	.076
Smoking, *n* (%)	968 (20.2)	330 (20.6)	320 (20)	318 (19.9)	.477
Drugs, *n* (%)					
β-blocker	522 (10.9)	176 (11.0)	164 (10.2)	182 (11.4)	.429
Statins	810 (16.8)	282 (17.3)	270 (16.9)	258 (16.1)	.063
HIF-PHI	944 (19.7)	337 (21.1)	317 (19.8)	290 (18.2)	.113
Iron	3632 (75.7)	1231 (76.9)	1206 (75.4)	1195 (74.7)	.326
ESAs (IU/kg. wk)	155.4 ± 42.9	168.6 ± 50.1	150.1 ± 47.3	131.3 ± 40.2	<.001
Diabetes, *n* (%)	1560 (32.5)	607 (37.9)	519 (32.5)	434 (27.1)	<.001
Hypertension, *n* (%)	3805 (79.3)	1279 (80.0)	1266 (79.2)	1260 (78.8)	.796
Cardiovascular history, *n* (%)	1330 (27.7)	549 (34.3)	440 (27.5)	341 (21.3)	<.001
Body mass index(kg/m^2^)	22.1 ± 3.9	21.8 ± 4.7	22.0 ± 3.3	22.2 ± 3.9	.098
Hemoglobin (g/L)	95.6 ± 24.8	80.7 ± 19.0	94.3 ± 21.2	111.9 ± 23.3	<.001
Neutrophil (10^9^/L)	4.3 ± 1.6	4.2 ± 1.6	4.3 ± 1.7	4.4 ± 1.7	.003
Lymphocyte (10^9^/L)	1.4 ± 0.6	1.0 ± 0.4	1.3 ± 0.4	1.7 ± 0.6	<.001
Monocyte (10^9^/L)	0.4 ± 0.3	0.4 ± 0.2	0.4 ± 0.2	0.5 ± 0.4	<.001
Platelet (10^9^/L)	193.1 ± 69.8	209.9 ± 76.4	192.5 ± 67.2	177.0 ± 61.1	<.001
AST (U/L)	21.8 ± 8.2	21.7 ± 9.1	21.5 ± 7.9	22.2 ± 8.3	.242
ALT (U/L)	22.3 ± 7.5	21.9 ± 7.8	22.1 ± 8.0	22.6 ± 7.3	.181
Albumin (g/L)	36.2 ± 6.3	33.4 ± 5.6	36.2 ± 5.0	39.0 ± 6.9	<.001
Triglyceride (mmol/L)	1.67 ± 0.37	1.66 ± 0.32	1.69 ± 0.23	1.68 ± 0.43	.190
Total cholesterol (mmol/L)	4.64 ± 1.33	4.58 ± 1.31	4.66 ± 1.43	4.69 ± 1.37	.064
LDL-c (mmol/L)	2.65 ± 0.99	2.62 ± 0.98	2.67 ± 1.02	2.66 ± 0.99	.324
HDL-c (mmol/L)	1.19 ± 0.39	1.17 ± 0.42	1.20 ± 0.38	1.21 ± 0.36	.345
Creatinine (umol/L)	781.9 (614.0, 1014.0)	768.0 (590.8, 1045.0)	800.0 (639.3, 1031.0)	776.2 (611.5, 984.0)	.099
eGFR (ml/min/1.73m^2^)	5.9 (4.3, 7.9)	5.9 (4.3, 8.2)	5.6 (4.3, 7.6)	6.0 (4.5, 7.8)	.161
Uric acid (μmol/L)	413.1 ± 113.5	419.3 ± 123.2	412.3 ± 110.3	407.9 ± 106.4	.059
Calcium (mmol/L)	2.14 ± 0.47	2.06 ± 0.71	2.15 ± 0.26	2.22 ± 0.26	<.001
Phosphate (mmol/L)	1.68 ± 0.55	1.79 ± 0.59	1.69 ± 0.54	1.56 ± 0.50	<.001
Intact parathyroid hormone (pg/mL)	230.3 (123.1, 414.3)	225.0 (124.5, 417.3)	227.1 (125.0, 399.8)	241.0 (119.9, 441.7)	.741
C-reactive protein (mg/L)	3.0 (1.1, 8.4)	3.8 (1.5, 10.3)	2.8 (1.1, 7.4)	2.3 (0.9, 6.0)	<.001

HIF-PHI.

ALT = alanine aminotransferase, AST = aspartate aminotransferase, eGFR = estimated glomerular filtration rate, ESAs = erythropoiesis stimulating agents, HDL-c = high-density lipoprotein cholesterol, HIF-PHI = hypoxia inducible factor-prolyl hydroxylase inhibitor, LDL-c = low-density lipoprotein cholesterol.

It is worth noting that there was no significant difference in HALP change (∆HALP) between Tertile groups [2.19 ± 0.68 (Tertile 1) vs 2.22 ± 0.94 (Tertile 2) vs 2.25 ± 0.83 (Tertile 3), *P* = .120].

### 3.2. Correlation between HALP and poor prognosis

**Figure 2.**
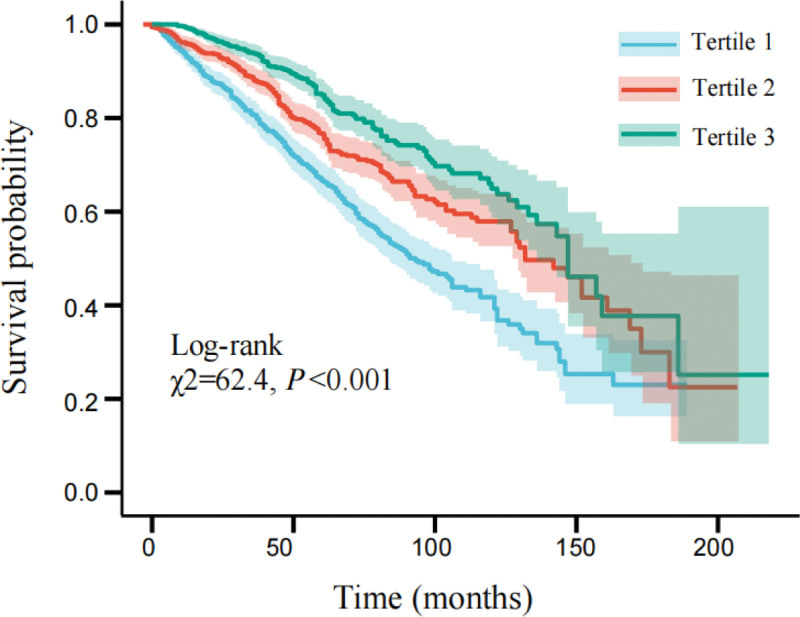
Kaplan–Meier curves of all-cause mortality in different hemoglobin, albumin, lymphocyte, and platelet (HALP).

The mean follow-up time of whole participants was 65 (interquartile range: 36, 96) months, and 1907 patients died at the end of follow-up, of which 1024 (53.6%) died from CVD. The results of Kaplan–Meier survival curve were demonstrated that the overall survival rate of patients in Tertile 3 of HALP was dramatically lower than that in Tertiles 1 and 2 (Log-rank test χ2 = 62.4, *P* < .001). (Fig. 2).

**Figure 3.**
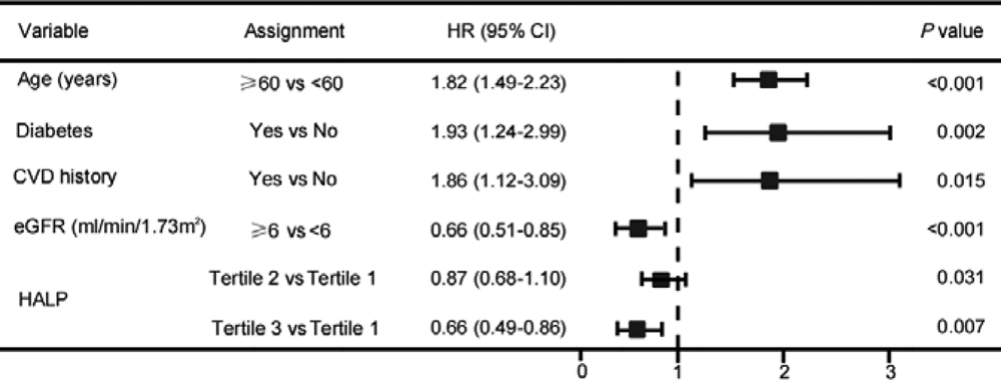
Forest plot of multivariate Cox analysis in all-cause mortality. CVD = cardiovascular disease, eGFR = estimated glomerular filtration rate, HALP = hemoglobin, albumin, lymphocyte, and platelet.

Depending on adjustment of multiple covariates model, the conclusions of the Cox analysis revealed that the increasing HALP was connected with decline exposure of all-cause death in continuous variables [hazard ratios (HR) = 0.96, 95% confidence intervals (CI): 0.95–0.97, *P* < .001], while in classified variables, the hazard of all-cause death in Tertile 3 was considerably lower than that in Tertile 1 (HR = 0.87, 95% CI: 0.68–1.10, *P* = .031; HR = 0.66, 95% CI: 0.49–0.86, *P* = .007, respectively) (Table 2). For other variables that influenced all-cause death in HD was presented in forest graph. (Fig. 3). There was, however, no significant association between ∆HALP and mortality (*P* = .142).

**Table 2:** Multivariate Cox regression analysis hemoglobin, albumin, lymphocyte, and platelet (HALP) and all-cause mortality.

	Crude Model		Model 1		Model 2	
	HR (95% CI)	*P* value	HR (95% CI)	*P* value	HR (95% CI)	*P* value
Continuous	0.97 (0.96–0.98)	<.001	0.98 (0.97–0.99)	<.001	0.96 (0.95–0.97)	<.001
Categories						
Tertile 1	1.0 (ref.)		1.0 (ref.)		1.0 (ref.)	
Tertile 2	0.66 (0.54–0.81)	<.001	0.85 (0.68–1.06)	.028	0.87 (0.68–1.10)	.031
Tertile 3	0.49 (0.39–0.61)	<.001	0.62 (0.44–0.80)	.002	0.66 (0.49–0.86)	.007
*P* for trend	<.001		.001		.003	

Model 1: was adjusted for age, gender, smoking, β-blocker, statins, ESAs, history of cardiovascular disease, hypertension and diabetes. Model 2: was adjusted for Model 1 + clinic factors (body mass index, blood lipid, uric acid, estimated glomerular filtration rate, calcium, phosphate, intact parathyroid hormone and C-reactive protein).

CI = confidence intervals, ESAs = erythropoiesis stimulating agents, HR = hazard ratios.

**Figure 4.**
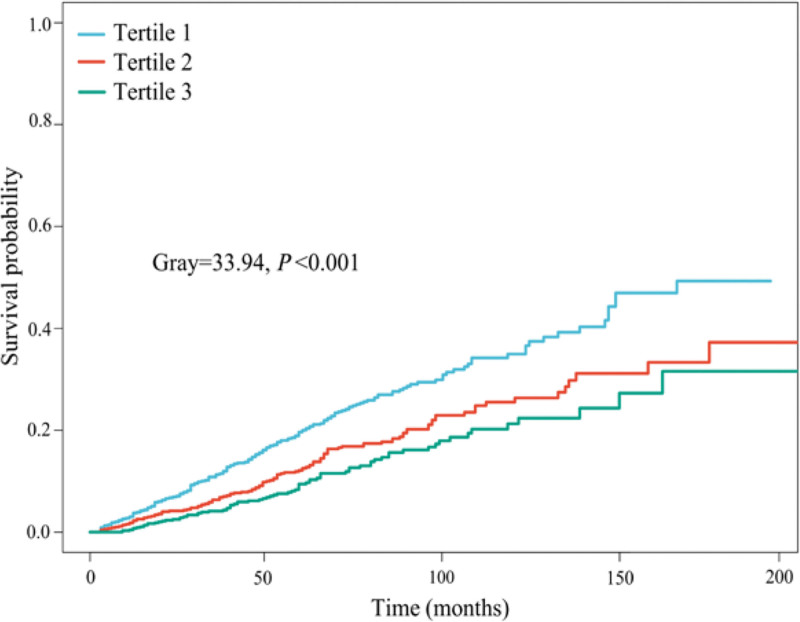
Relationship in diverse hemoglobin, albumin, lymphocyte, and platelet (HALP) and cardiovascular disease (CVD) mortality (Fine-Gray competing risk model).

Further analysis, the results of Fine-Gray competitive risk model revealed that the decrease of HALP was also related to the increase of CVD mortality (Gray = 33.94, *P* < .001) (Fig. 4). The corrected CVD death risk ratio of the Tertile 3 of HALP is significantly lower than that of the Tertile 1 (sub-distribution hazard ratio = 0.51, 95% CI: 0.34–0.80, *P* = .005) (Table 3).

**Table 3:** Sub-distribution hazard ratio (SHR) about hemoglobin, albumin, lymphocyte, and platelet (HALP) and cardiovascular disease (CVD) mortality.

	Crude Model		Model 1		Model 2	
	SHR (95% CI)	*P* value	SHR (95% CI)	*P* value	SHR (95% CI)	*P* value
Tertile 1	1.0 (ref.)		1.0 (ref.)		1.0 (ref.)	
Tertile 2	0.59 (0.46–0.76)	<.001	0.66 (0.52–0.89)	.004	0.74 (0.53–1.04)	.036
Tertile 3	0.37 (0.28–0.49)	<.001	0.39 (0.28–0.54)	<.001	0.51 (0.34–0.80)	.005

Model 1: was adjusted for age, gender, smoking, β-blocker, statins, ESAs, history of cardiovascular disease, hypertension and diabetes. Model 2: was adjusted for Model 1 + clinic factors (body mass index, blood lipid, uric acid, estimated glomerular filtration rate, calcium, phosphate, intact parathyroid hormone and C-reactive protein).

CI = confidence intervals, ESAs = erythropoiesis stimulating agents, SHR = sub-distribution hazard ratio.

**Figure 5.**
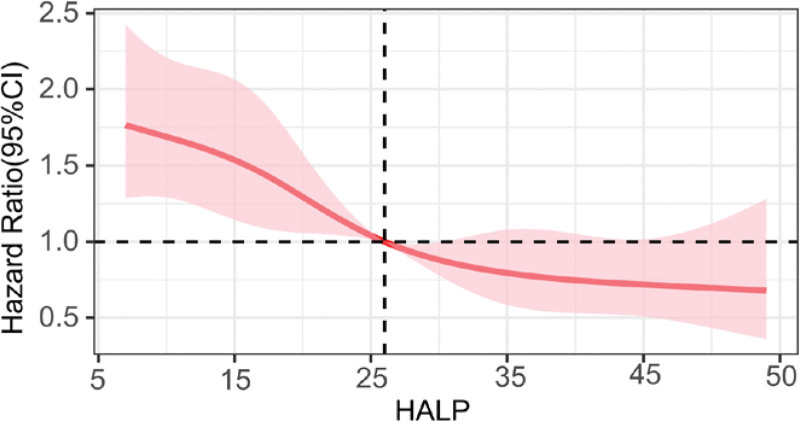
Restricted cubic splines of hemoglobin, albumin, lymphocyte, and platelet (HALP) and all-cause mortality.

Meanwhile, the multivariate adjusted restricted cubic splines revealed that there was a linear correlation between HALP and all-cause death in HD (*P* for non-linearity = 0.436), showing a trend that the dwindle HALP the more advanced hazard of death (Fig. 5).

### 3.3. The forecast value of HALP for mortality

**Figure 6.**
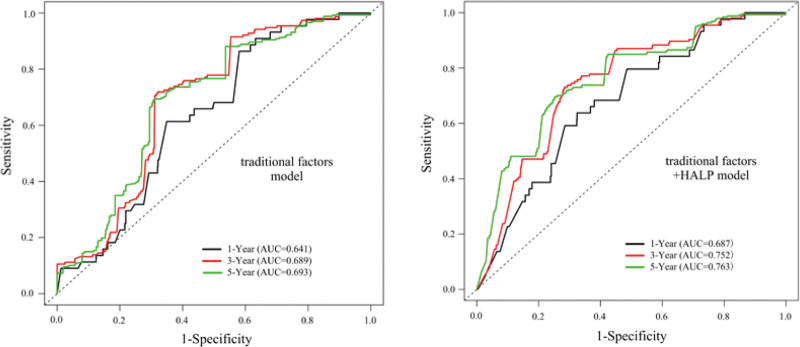
Time-dependent receiver operating characteristic (ROC) of hemoglobin, albumin, lymphocyte, and platelet (HALP) and all-cause mortality.

The combination of previously obtained factors (age, history of CVD, diabetes, and eGFR) affecting the prognosis of all-cause mortality and common controversial HD risk factors (gender, smoking, β-blocker, statins, erythropoiesis stimulating agents, hypertension, total cholesterol, high-density lipoprotein cholesterol, low-density lipoprotein cholesterol, CRP, calcium, phosphate, and iPTH) was named the traditional factors model, when the HALP was added to this model, named the traditional factors + HALP model. Time-dependent receiver operating characteristic curve analysis exhibited that the area under curve of the traditional factors model for all-cause mortality was 0.641 at 1 year, 0.689 at 3 years, and 0.693 at 5 years, while the traditional factors + HALP model was 0.687 at 1 year, 0.752 at 3 years, and 0.763 at 5 years, all *P* < .001 (Fig. 6).

Furthermore, statistical analysis results indicated that the C-index of traditional factors model was 0.71 (0.66–0.75), *P* < .001; and traditional factors + HALP model was 0.76 (0.72–0.79), *P* < .001, (delta C-statistics: 0.05). The net reclassification improvement and integrated discrimination improvement were 0.09, *P* = .001; 0.33, *P* < .001, respectively (Table 4). These statistical results suggested that the traditional factors + HALP model had superior predictive value than the traditional factors model.

**Table 4:** C-index, NRI, and IDI for HALP predictive value of all-cause death.

Variable	C-index		delta C-statistics		NRI		IDI	
	Estimate (95% CI)	*P* value	Estimate (95% CI)	*P* value	Estimate (95% CI)	*P* value	Estimate (95% CI)	*P* value
Traditional factors model	0.71 (0.66–0.75)	<.001	Reference		Reference		Reference	
Traditional factors + HALP model	0.76 (0.72–0.79)	<.001	0.05 (0.01–0.08)	<.001	0.09 (0.05–0.12)	.001	0.33 (0.23–0.43)	.002

Traditional factors = age, gender, smoking, β-blocker, statins, ESAs, history of cardiovascular disease, hypertension and diabetes, total cholesterol, high-density lipoprotein cholesterol, low-density lipoprotein cholesterol, C-reactive protein, calcium, phosphate, intact parathyroid hormone and estimated glomerular filtration rate.

CI = confidence intervals, ESAs = erythropoiesis stimulating agents, HALP = hemoglobin, albumin, lymphocyte, and platelet, IDI = integrated discrimination improvement, NRI = net reclassification improvement.

## 4. Discussion

The source of this research was a multi-center with a long follow-up period and a large sample of database. To our knowledge, this was a initial finding that decreased baseline HALP values were distinctly correlated with inferior outcome under HD (cubic spline curves indicated a negative linear relationship with risk of all-cause death). Multiple statistical analyses of study confirmed high risk for CVD and all-cause mortality as well. Cox analysis revealed an isolated association between HALP and undesirable outcome, after adjusting for multiple covariates, indicating that it was independent of traditional risk factors (according to the HALP tertiles, patients with the lowest score had a 34% increased risk of all-cause death relative to the highest, after adjustment). Additionally, we have validated that adding HALP values to traditional risk factor models could boost the forecast competence of all-cause mortality. In brief, this data was presented to demonstrate that dwindle HALP values could be a risk factor for poor prognosis in HD patients.

Previous studies have point out that nutrition and inflammatory are convincingly related to the outcomes of HD patients.^[14,15]^ Anemia can contribute to ventricular hypertrophy, aggravating heart failure and myocardial ischemia, resulting in low immunity, increasing infection and other adverse complications, and connecting with the occurrence of CVD events ultimately, low quality of life, cognitive impairment and mortality.^[16]^ Hypoalbuminemia is a manifestation of malnutrition, which weakens the immune response and makes infection more likely and also associated with inflammation in the body.^[17]^ Inflammation can trigger embolism, where platelets involved in conglutination, enhanced reaction and polymerization. Simultaneously, in the process of inflammation clearance and ameliorating, immune cells play a significant role.^[18]^ Researches have found that high platelet-lymphocyte ratio is associated with arteriovenous fistula stenosis and thrombosis in HD patients.^[19]^ In consequence, HALP has a strong biological basis as an indicator integrating the interactions between nutrition, inflammation, platelet function, and immune function.

Based on the calculation formula of HALP, lower values means more potential inflammation, poorer immune response, higher risk of blood clots, and more severe malnutrition. At the same time, through the data of this study, it was found that lower HALP also seemed to be concerned in hypocalcemia and hyperphosphatemia, which are the manifestations of chronic kidney disease-mineral and bone disorder.^[20]^ Therefore, it makes sense that low HALP has relation to a poor prognosis in HD. In fact, recently, it has been reported that HALP can indicate the malnutrition and systemic inflammatory state of chronic diseases, and it turns out to be a relevant outcome symbol for different tumors.^[9–13]^ Interestingly, there are some improved indicators based on blood laboratory tests, such as Neutrophil-Lymphocyte Ratio and Monocyte-lymphocyte ratio,^[21,22]^ which are often mentioned in the analysis of the appraisal of nutritional and inflammatory condition in dialysis subjects.

Among the traditional prognostic factors of HD, there are often inconsistent conclusions about eGFR, calcium, phosphorus, and iPTH. It is a simple utility that CRP or lipid variables reflect the body, while β-blockers, CVD history, and diabetes are closely related to the patient’s past history and fail to highlight the characteristics of hemodialysis. Micro-inflammatory factors, such as tumor necrosis factor-α or interleukin-6, cannot be routinely measured. However, the HALP is a simple, effective and low-cost assessment indicator for a more comprehensive assessment of the inflammatory-nutritional status of HD patients. When used alone, it can reflect the inflammation-nutritional status of patients and predict the poor prognosis of HD patients, while combined with traditional risk factors, this effect was significantly increased.

There were some inadequacies in our work. Primarily, as a retrospective research, election bias was inevitable and causality could not be clearly defined at this stage. Secondly, although there were many confounding factors included in correction, dynamic factors such as detailed drug prescription changes, maintenance of dialysis vascular access, and fluctuations in dialysis adequacy could not be completely analyzed due to incomplete data. Ultimately, the optimal diagnostic HALP values remain unclear. Consequently, deeper prospective studies are necessary to validate the results thoroughly in following investigation.

## 5. Conclusion

Our analysis demonstrated a negative association between baseline HALP and risk of all-cause death and CVD death in HD patients, suggesting that HALP values before dialysis may be a reliable indicator of poor prognosis.

## Acknowledgments

The authors would to be grateful for the whole of participants about their dedication in this paper.

## Author contributions

**Conceptualization:** Fengping Zhang, Luohua Li, Le Yu.

**Data curation:** Fengping Zhang, Luohua Li.

**Formal analysis:** Fengping Zhang, Luohua Li.

**Investigation:** Fengping Zhang, Luohua Li, Taotao Shi, Yu Liu, Jun Xie, Le Yu.

**Methodology:** Fengping Zhang, Luohua Li, Le Yu.

**Project administration:** Le Yu.

**Resources:** Luohua Li, Taotao Shi, Yu Liu, Jun Xie, Le Yu.

**Software:** Luohua Li.

**Supervision:** Luohua Li, Le Yu.

**Writing – original draft:** Fengping Zhang.

**Writing – review & editing:** Le Yu.

## References

[1] Bello AK Okpechi IG Osman MA. Epidemiology of haemodialysis outcomes. Nat Rev Nephrol. 2022;18:378–95. 35194215 10.1038/s41581-022-00542-7 PMC8862002

[2] Tong A Manns B Hemmelgarn B. Establishing core outcome domains in hemodialysis: report of the standardized outcomes in nephrology-hemodialysis (SONG-HD) consensus workshop. Am J Kidney Dis. 2017;69:97–107. 27497527 10.1053/j.ajkd.2016.05.022 PMC5369351

[3] Gansevoort RT Correa-Rotter R Hemmelgarn BR. Chronic kidney disease and cardiovascular risk: epidemiology, mechanisms, and prevention. Lancet. 2013;382:339–52. 23727170 10.1016/S0140-6736(13)60595-4

[4] Cozzolino M Mangano M Stucchi A. Cardiovascular disease in dialysis patients. Nephrol Dial Transplant. 2018;33(suppl_3):iii28–34. 30281132 10.1093/ndt/gfy174 PMC6168816

[5] Sautenet B Tong A Williams G. Scope and consistency of outcomes reported in randomized trials conducted in adults receiving hemodialysis: a systematic review. Am J Kidney Dis. 2018;72:62–74. 29475768 10.1053/j.ajkd.2017.11.010

[6] Htay H Bello AK Levin A. Hemodialysis use and practice patterns: an international survey study. Am J Kidney Dis. 2021;77:326–335.e1. 32800843 10.1053/j.ajkd.2020.05.030

[7] Güç ZG Alacacioğlu A Kalender ME. HALP score and GNRI: simple and easily accessible indexes for predicting prognosis in advanced stage NSCLC patients. The İzmir oncology group (IZOG) study. Front Nutr. 2022;9:905292. 36061883 10.3389/fnut.2022.905292 PMC9437940

[8] Xu M Chen L Hu Y. The HALP (hemoglobin, albumin, lymphocyte, and platelet) score is associated with early-onset post-stroke cognitive impairment. Neurol Sci. 2023;44:237–45. 36192653 10.1007/s10072-022-06414-z

[9] Peng D Zhang CJ Gong YQ. Prognostic significance of HALP (hemoglobin, albumin, lymphocyte and platelet) in patients with bladder cancer after radical cystectomy. Sci Rep. 2018;8:794. 29335609 10.1038/s41598-018-19146-y PMC5768698

[10] Lou C Jin F Zhao Q. Correlation of serum NLR, PLR and HALP with efficacy of neoadjuvant chemotherapy and prognosis of triple-negative breast cancer. Am J Transl Res. 2022;14:3240–6. 35702128 PMC9185079

[11] Kocaoglu S Alatli T. The efficiency of the HALP score and the modified HALP score in predicting mortality in patients with acute heart failure presenting to the emergency department. J Coll Physicians Surg Pak. 2022;32:706–11. 35686400 10.29271/jcpsp.2022.06.706

[12] Tian M Li Y Wang X. The hemoglobin, albumin, lymphocyte, and platelet (HALP) score is associated with poor outcome of acute ischemic stroke. Front Neurol. 2021;11:610318. 33510706 10.3389/fneur.2020.610318 PMC7835486

[13] Peng D Zhang CJ Tang Q. Prognostic significance of the combination of preoperative hemoglobin and albumin levels and lymphocyte and platelet counts (HALP) in patients with renal cell carcinoma after nephrectomy. BMC Urol. 2018;18:20. 29544476 10.1186/s12894-018-0333-8 PMC5855974

[14] Balbino KP Juvanhol LL Wendling AL. Dietary inflammatory index and mortality in hemodialysis patients by path analysis approach (NUGE-HD study). Nutrition. 2021;89:111239. 33930786 10.1016/j.nut.2021.111239

[15] Sahathevan S Khor BH Ng HM. Understanding development of malnutrition in hemodialysis patients: a narrative review. Nutrients. 2020;12:3147. 33076282 10.3390/nu12103147 PMC7602515

[16] Gluba-Brzózka A Franczyk B Olszewski R. The influence of inflammation on anemia in CKD patients. Int J Mol Sci. 2020;21:725. 31979104 10.3390/ijms21030725 PMC7036805

[17] Meijers BK Bammens B Verbeke K. A review of albumin binding in CKD. Am J Kidney Dis. 2008;51:839–50. 18436096 10.1053/j.ajkd.2007.12.035

[18] Cao C Yao Y Zeng R. Lymphocytes: versatile participants in acute kidney injury and progression to chronic kidney disease. Front Physiol. 2021;12:729084. 34616308 10.3389/fphys.2021.729084 PMC8488268

[19] Sarioglu O Capar AE Belet U. Relationship of arteriovenous fistula stenosis and thrombosis with the platelet-lymphocyte ratio in hemodialysis patients. J Vasc Access. 2020;21:630–5. 31884875 10.1177/1129729819894113

[20] Kidney Disease: Improving Global Outcomes (KDIGO) CKD-MBD Work Group. KDIGO clinical practice guideline for the diagnosis, evaluation, prevention, and treatment of chronic kidney disease-mineral and bone disorder (CKD-MBD). Kidney Int Suppl. 2009;113:S1–130. 10.1038/ki.2009.188 19644521

[21] Bołtuć K Bociek A Dziugieł R. Neutrophil-lymphocyte ratio (NLR) reflects myocardial inhomogeneities in hemodialyzed patients. Mediators Inflamm. 2020;2020:6027405. 32963494 10.1155/2020/6027405 PMC7486637

[22] Xiang F Chen R Cao X. Monocyte/lymphocyte ratio as a better predictor of cardiovascular and all-cause mortality in hemodialysis patients: a prospective cohort study. Hemodial Int. 2018;22:82–92. 28403540 10.1111/hdi.12549

